# An integrative survival analysis and a systematic review of the cerebellopontine angle glioblastomas

**DOI:** 10.1038/s41598-023-30677-x

**Published:** 2023-03-17

**Authors:** Nebojsa Lasica, Kenan Arnautovic, Tomita Tadanori, Petar Vulekovic, Dusko Kozic

**Affiliations:** 1 Clinic of Neurosurgery, University Clinical Center of Vojvodina, Hajduk Veljkova 1-9, 21000 Novi Sad, Serbia; 2 Faculty of Medicine, University of Novi Sad, Novi Sad, Serbia; 3 Semmes Murphey Clinic, Memphis, TN USA; 4 Department of Neurosurgery, University of Tennessee Health Science Center, Memphis, TN USA; 5 Division of Pediatric Neurosurgery, Ann & Robert H. Lurie Children’s Hospital of Chicago and Northwestern University Feinberg School of Medicine, Chicago, IL USA; 6 Center for Diagnostic Imaging, Oncology Institute of Vojvodina, Sremska Kamenica, Serbia

**Keywords:** Surgical oncology, Cancer imaging, CNS cancer

## Abstract

Glioblastomas presenting topographically at the cerebellopontine angle (CPA) are exceedingly rare. Given the specific anatomical considerations and their rarity, overall survival (OS) and management are not discussed in detail. The authors performed an integrative survival analysis of CPA glioblastomas. A literature search of PubMed, Scopus, and Web of Science databases was performed per PRISMA guidelines. Patient data including demographics, clinical features, neuroimaging, management, follow-up, and OS were extracted. The mean age was 39 ± 26.2 years. The mean OS was 8.9 months. Kaplan–Meier log-rank test and univariate Cox proportional-hazards model identified hydrocephalus (log-rank, p = 0.034; HR 0.34; 95% CI 0.12–0.94; p = 0.038), chemotherapy (log-rank, p < 0.005; HR 5.66; 95% CI 1.53–20.88; p = 0.009), and radiotherapy (log-rank, p < 0.0001; HR 12.01; 95% CI 3.44–41.89; p < 0.001) as factors influencing OS. Hydrocephalus (HR 3.57; 95% CI 1.07–11.1; p = 0.038) and no adjuvant radiotherapy (HR 0.12; 95% CI 0.02–0.59; p < 0.01) remained prognostic on multivariable analysis with fourfold and twofold higher risk for the time-related onset of death, respectively. This should be considered when assessing the risk-to-benefit ratio for patients undergoing surgery for CPA glioblastoma.

## Introduction

Glioblastomas represent around 15% of all intracranial tumors and account for more than 50% of primary and other central nervous system (CNS) gliomas^1^. They are usually located in the supratentorial region, although they may develop at any location within the CNS^2^. Nevertheless, they rarely develop in the posterior fossa, particularly in the adult population group, where they make up approximately 1% of all glioblastomas^3^.

Brainstem gliomas have a stereotypical growth pattern due to anatomical barriers, greatly influenced by pia, decussations zones, and brainstem projections, which usually make them protrude dorsally^4^. Proportionately, glioblastomas can develop as exophytic extensions to the cerebellopontine angle (CPA) from the adjacent brainstem or the cerebellum, and may also develop from the root entry zone (REZ) of the cranial nerves^5^. CPA is one of the most complex anatomical regions of the CNS, with a plethora of lesions that may arise from various tissues of CPA, embryological remnants, and extensions from adjacent structures, including petrous bone, brainstem, and ventricles^6^. Imaging-wise these tumors may exert similar characteristics, and topographically appear within the CPA. Diagnostic confidence interval for CPA glioblastomas may be enhanced through the identification of abnormal imaging characteristics and growth pattern on sequential imaging, unexpected for the common CPA lesions.

Maximal surgical cytoreduction with adjuvant treatment remain standard in the treatment of glioblastoma^7^. Considering specific anatomical relationships, neurosurgeons must determine best treatment strategy that can be applied for CPA glioblastomas based on previous experience and predictors that influence survival.

In this communication, we present our illustrative case of CPA glioblastoma and review pertinent literature to carry out an integrative survival analysis of reported cases and the relationship between the various factors and overall survival (OS).

## Results

### Integrated cohort

The search strategy revealed a total of 124 articles for evaluation. After removing duplicate articles, inclusion criteria were applied to 101 titles and abstracts of articles. Twenty-seven articles underwent full-text analysis. One article was included from the manual search of references. Overall, 26 articles describing CPA glioblastoma satisfied the inclusion criteria. Pooled cases and a case from our institution were included in the final integrated cohort, consisting of 30 patients for analysis. A PRISMA flow diagram shown (Supplementary Fig. 1) depicts the search strategy.

### Patients and tumor characteristics

The mean age at presentation was 39 years. Eleven (36.7%) patients belonged to the pediatric and 19 (63.3%) to the adult group. Slight male (60%) predominance was observed. The mean tumor size was 37.7 ± 15.7 mm. Presenting symptoms included disequilibrium (50.3%), hearing loss (16.7%), and signs of increased intracranial pressure included headache, nausea or vomiting, facial weakness, facial numbness, and visual changes (each 13.3%). The mean duration of symptoms was 3 months. Signs of hydrocephalus on admission were noted in 11 patients (36.7%).

CPA glioblastomas were most commonly reached through the retrosigmoid approach (76%). Other approaches included: combined subtemporal and retrosigmoid, subtemporal, translabyrinthine, far lateral, and suboccipital. Tumors originated from the cranial nerve REZ (30.0%), most commonly from the cranial nerve VIII (Supplementary Table 1); the remainder were secondary exophytic and originated from the pons (26.7%) and the cerebellum (36.7%). Twenty-nine (96.7%) patients underwent surgery, with GTR achieved in 4 (13.3%), STR in 20 (66.7%), and biopsy in 5 (16.7%) patients. Thirteen (43.3%) patients received postoperative radiotherapy and chemotherapy, and 7 (23.3%) received radiotherapy alone. The mean follow-up was 12 months (range 3–24 months). No cerebrospinal fluid (CSF) dissemination within the neuroaxis or through a shunt was observed in this cohort. A detailed overview of baseline patient and tumor characteristics are shown in Table 1.

**Table 1:** Patient demographics and tumor characteristics.

Variable	Value
Mean age (range), years	39.3 (3–79)
Sex, n (%)	
Male	18 (60.7)
Female	11 (39.3)
Presenting symptom, n (%)	
Disequilibrium	16 (50.3)
Hearing loss	5 (16.7)
HA/N/V	4 (13.3)
Facial weakness	4 (13.3)
Facial numbness	4 (13.3)
Visual changes	4 (13.3)
Hemiparesis	3 (10.0)
Bulbar symptoms	1 (3.3)
Facial pain	1 (3.3)
Otalgia	1 (3.3)
Tinnitus	1 (3.3)
Symptom duration, months	
Mean ± SD	3.2 ± 6.7
Tumor origins, n (%)	
Cranial nerve	9 (30.0)
Pons	8 (26.7)
Cerebellum	11 (36.7)
NOS	3 (10.0)
Mean tumor size (range), mm	37.7 (8–78)
Hydrocephalus, n (%)	11 (36.7)
Surgery type, n (%)	
Biopsy	5 (16.7)
STR	20 (66.7)
GTR	4 (13.3)
None	1 (3.3)
Treatment modality, n (%)	
Surgery + RT + CT	13 (43.3)
Surgery + RT	7 (23.3)
Surgery + CT	0 (0.0)
Surgery	9 (30.0)
None	1 (3.3)
Follow-up	
Mean ± SD	12.0 ± 6.7
Overall survival, months	
Mean ± SD	8.9 ± 8.0

#### Survival analysis

**Figure 1:**
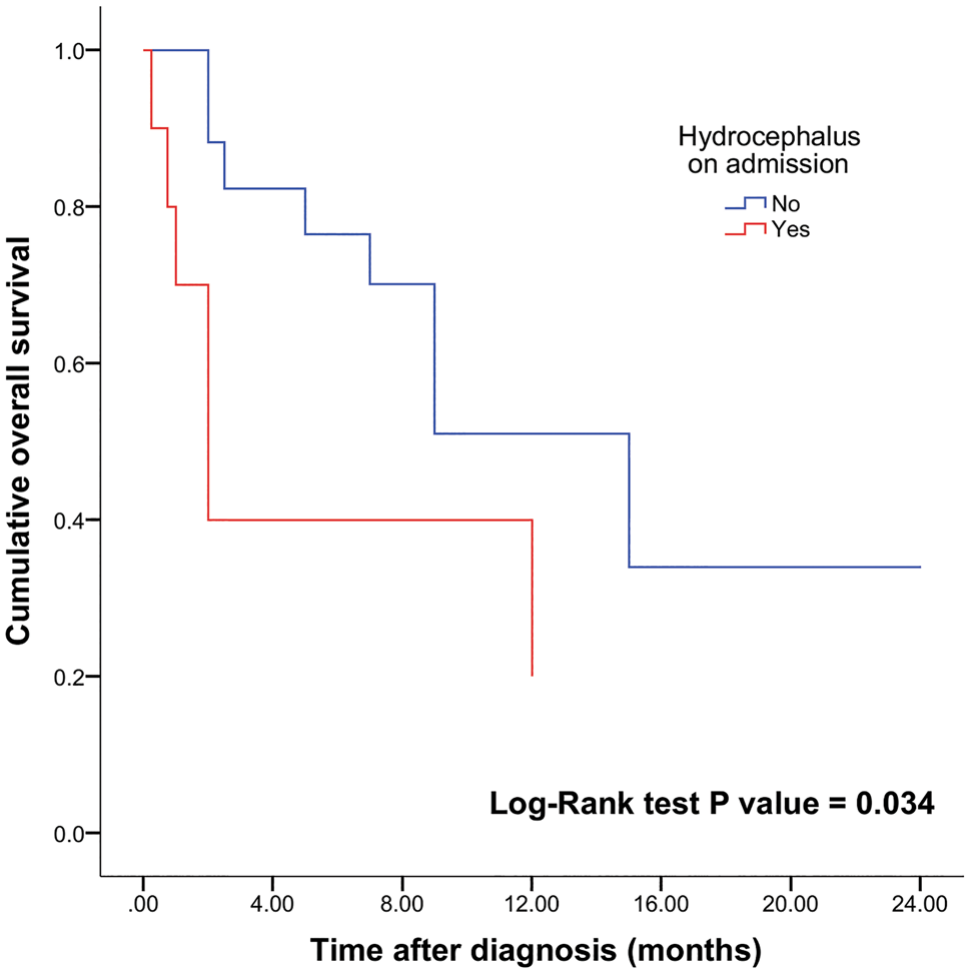
Kaplan–Meier plot of OS based on presence (n = 11) or absence (n = 19) of hydrocephalus in patients with CPA glioblastoma (log-rank test alpha level was 0.05). Patients with no hydrocephalus on admission had a mean OS 18.4 months compared with 5.6 months in patients with hydrocephalus on admission.

**Figure 2:**
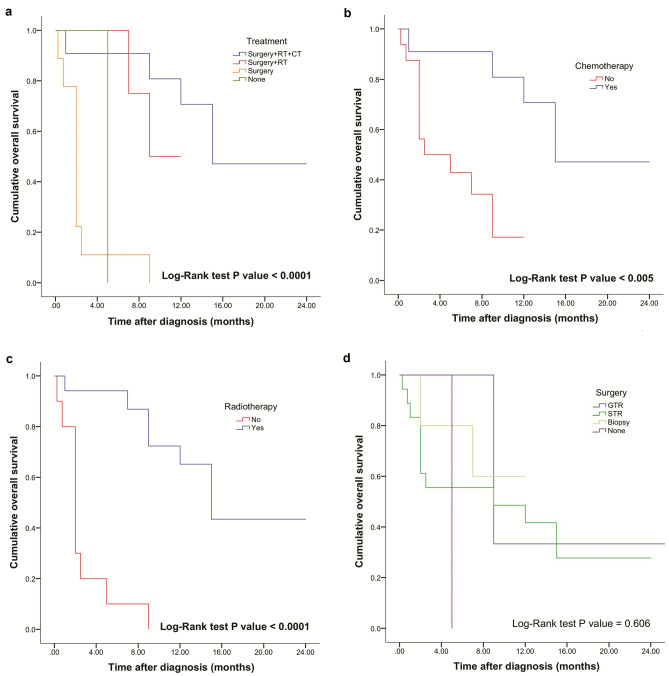
Comparison of OS based on treatment modalities (**a**) with patient subgroups receiving surgery and complete adjuvant treatment (n = 13), surgery and adjuvant radiation treatment (n = 7), surgery alone (n = 9), and no treatment (n = 1). Kaplan–Meier plot of chemotherapy (n = 13), and no adjuvant chemotherapy (n = 17) subgroups (**b**), and patients with CPA glioblastoma receiving radiation treatment (n = 20), and with no adjuvant radiotherapy (n = 10) (**c**). OS based on surgery type (**d**) with subgroup of patients that underwent GTR (n = 4), STR (n = 20), biopsy (n = 5), and no surgery (n = 1). Log-rank test alpha level was set to 0.05. Statistically longer survival was observed in patients receiving surgery with complete postoperative adjuvant treatment, postoperative chemotherapy, and radiation treatment.

Results from univariable and multivariable analysis using the Cox proportional-hazards model are shown in Table 2. Using univariable analysis, the presence of hydrocephalus on admission appeared to be a significant factor associated with mortality (HR 0.34; 95% CI 0.12–0.94; p = 0.038). Figure 1 reveals the Kaplan–Meier curve that illustrates a statistically significant separation of curves according to the presence of hydrocephalus (log-rank test, p = 0.024). Figure 2a demonstrates the combination of treatment modalities on OS. Other factors associated with survival found on univariable analysis were adjuvant chemotherapy (HR 5.66; 95% CI 1.53–20.88; p = 0.009) and radiotherapy (HR 12.01; 95% CI 3.44–41.89; p < 0.001) as demonstrated by Fig. 2b,c.

**Table 2:** Cox proportional hazard regression analysis of factors associated with mortality in CPA glioblastomas.

Variables	Univariable analysis			Multivariable analysis		
	HR	95% CI	p value	HR	95% CI	p value
Age						
Adult	1.0	1.0	1.0			
Pediatric	1.95	0.69–5.45	0.204			
Gender						
Female	1.0	1.0	1.0			
Male	0.74	0.25–2.24	0.599			
Hydrocephalus on admission						
Present	1.0	1.0	1.0			
Absent	2.94	1.06–8.33	**0.038**	3.57	1.07–11.1	**0.038**
Tumor origins						
Exophytic	1.0	1.0	1.0			
Nerve root entry zone	0.92	0.29–2.97	0.89			
Tumor size						
< 3 cm	1.0	1.0	1.0			
> 3 cm	2.38	0.63–8.99	0.200			
Surgery						
STR or biopsy	1.0	1.0	1.0			
GTR	0.70	0.16–3.13	0.64			
Adjuvant treatment						
Chemotherapy	0.18	0.04–0.65	**0.009**	0.44	0.07–2.86	0.393
Radiation therapy	0.08	0.02–0.29	** < 0.001**	0.12	0.02–0.59	** < 0.01**

Boldface type indicates statistical significance (p < 0.05).

The type of surgery did not influence the OS on Kaplan–Meier analysis (Fig. 2d); however, GTR showed longer mean OS (17.3 months) compared to STR (10.9 months) and biopsy only (9 months). No significant association was found between age group, sex, tumor origin, and size. Even so, it is interesting to note that the pontine origin was associated with the shortest mean OS (7.3 months) compared with the cranial nerve REZ (12.1 months) and cerebellum (18.4 months), and the influence of tumor size on mean OS (< 3 cm, 14.3 months vs > 3 cm 9.9 months). Furthermore, pediatric age group displayed shorter mean OS (5.9 months) compared with adults (17.9 months) (Supplementary Fig. 2).

**Figure 3:**
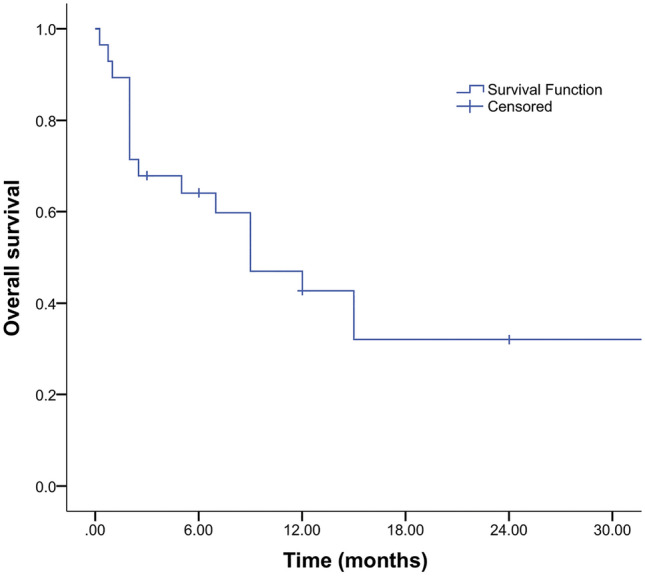
Kaplan–Meier curve showing OS survival in patients with CPA glioblastoma in our cohort.

The multivariable Cox proportional-hazards model confirmed the influence of hydrocephalus on OS (HR 3.57; 95% CI 1.07–11.1; p = 0.038) after adjusting for other variables; patients with hydrocephalus had an almost fourfold higher risk for the time-related onset of death. Similarly, patients not receiving adjuvant radiotherapy were prone and had an almost twofold higher risk of death (HR 0.12; 95% CI 0.02–0.59; p < 0.01) compared with patients with adjuvant radiotherapy. On the other hand, when adjusting to other factors, chemotherapy was no longer a significant risk factor for time-related onset of death (HR 0.44; 95% CI 0.07–2.86; p = 0.393). The Kaplan–Meier curve was created for OS (Fig. 3). For patients with CPA glioblastomas, cumulative survival estimated at 3, 6, 12, 18 and 24 months was 67.8%, 60.7%, 39.3%, 10.7%, and 10.7%, respectively; the overall death rate was highest in the first 3 months at 32.2%.

### Immunohistochemistry

Immunohistochemistry information was available in 21 (70.0%) patient. While there were some apparent differences in the percentage of positivity of some immunomarkers, the used markers were not uniform in each report (Supplementary Table 2). Most consistently reported were GFAP (50.0%), p53 (20.0%), and S100 (20.0%). No clear differences were observed between secondary exophytic and nerve REZ in immunomarkers positivity.

### Neuroimaging

Two (6.6%) CPA glioblastomas were located bilaterally. The remainder were located on the left (33.3%) and the right side (56.7%). Tumors most commonly showed a low-intensity signal on T1-weighted (91.7%), and a high-intensity signal on T2-weighted images (76.5%). On administration of contrast, they showed heterogeneous (60.0%), homogenous (16.0%), and peripheral (8.0%) enhancement. Four (16.0%) tumors appeared as ring lesions. Extension to the internal acoustic canal (IAC) was noted in 7 (23.3%) patients.

Proton magnetic resonance (MR) spectroscopy findings were available in 7 patients. Single and multivoxel spectroscopic data showed the most usual pattern of increased choline levels relative to *N*-acetyl aspartate. In addition, a relative decrease of creatine compared with choline levels was also reported. One study reported increased lactate on MR spectroscopy.

According to the available neuroimaging characteristics from reported cases, preoperative differential diagnoses included peripheral nerve sheath tumor (PNST) in 58.8% of cases (out of which 17.6% were suspected to be malignant peripheral nerve sheath tumor [MPNST]), meningioma (35.3%), both low-grade and high-grade glioma (35.3%), and metastases (17.6%). The remaining cases (41.2%) belonged to the group of less frequent differential diagnoses, including atypical teratoid rhabdoid tumor (ATRT), brain abscess, lymphoma, meningitis, neurosarcoidosis, primitive neuroectodermal tumor (PNET), and tuberculoma.

#### Illustrative case

**Figure 4:**
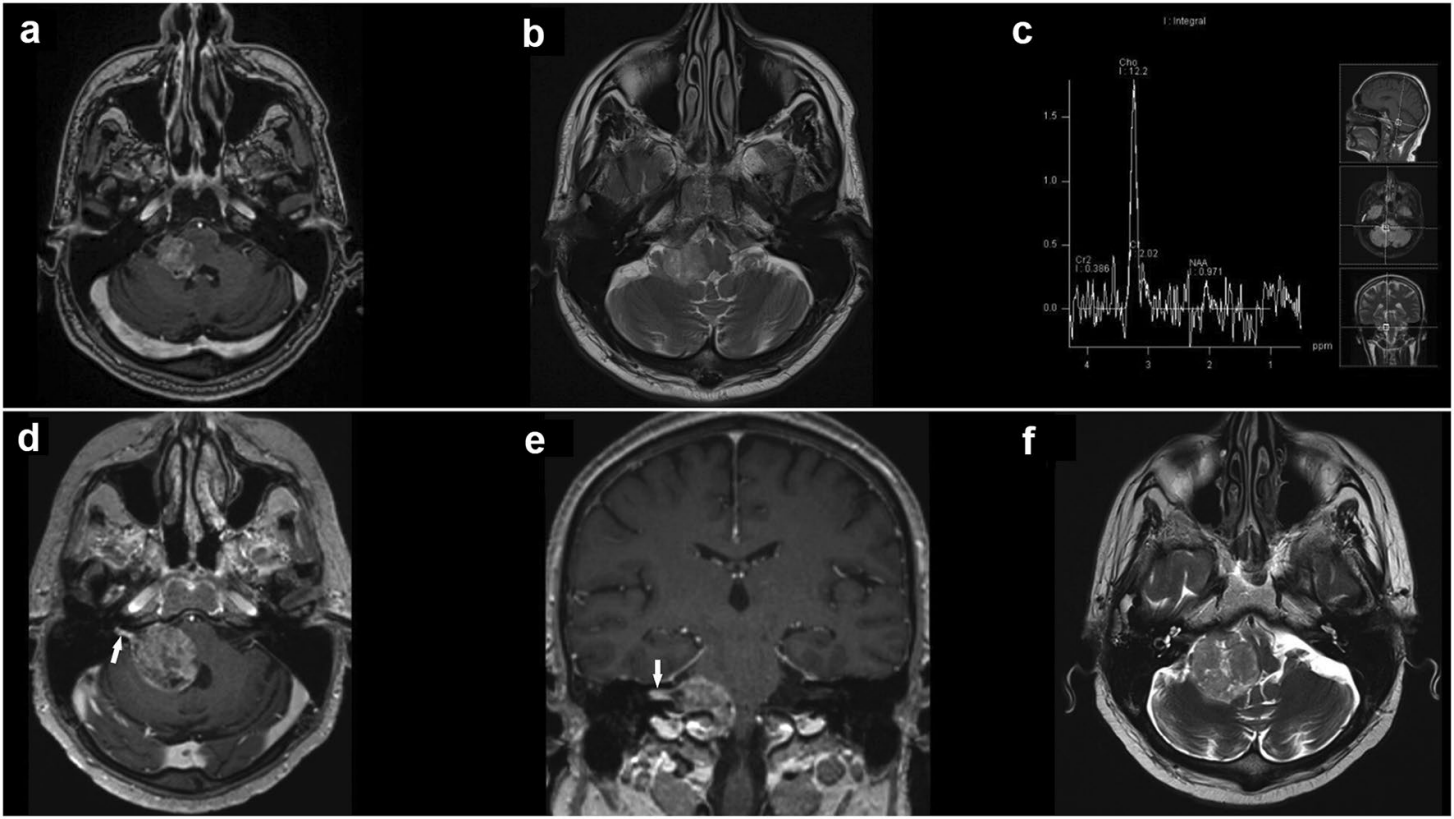
T1-weighted gadolinium-enhanced magnetization-prepared rapid gradient-echo MRI sequence of the brain in the axial plane (**a**) demonstrates a well-defined extraaxial solid mass of approximately 26 × 23 × 21 mm in the right CPA. T2-weighted MRI turbo spin-echo sequence of the brain in the axial plane (**b**) revealed peritumoral edema involving the right cerebellar peduncle and compressive effect on the brainstem, the fourth ventricle, and the right foramen of Luschka. Single-voxel MR spectroscopy of the CPA lesion (**c**) reveals elevated choline concentration, with no other metabolites. Follow-up T1-weighted gadolinium-enhanced MRI in the axial (**d**) and coronal planes (**e**) reveal marked enlargement of the tumor with extension to the IAC (arrow). Axial T2-weighted MRI (**f**) shows further expansion of the tumor mass to 35 × 34 × 33 mm and more pronounced compression on the lateral aspect of the brainstem and the fourth ventricle.

A male patient in his 50 s presented with a 6-month history of progressive right-sided facial weakness and numbness, and gradual hearing loss in the right ear before admission. The patient’s past medical and family history was unremarkable. Neurological examination revealed hearing loss and peripheral facial nerve palsy, and anesthesia on the right side. Imaging revealed a well-defined extraaxial heterogeneous solid mass in the right CPA. Radiological findings and neurometabolic profile on MR spectroscopy were most likely consistent with vestibular schwannoma (VS) (Fig. 4a–c). After discussing treatment options, including observation, stereotactic radiosurgery (SRS), and microsurgical resection the patient opted for observation. Interval follow-up MRI acquired at 5 months demonstrated marked enlargement of the tumor (Fig. 4d–f). Due to the progression in a short time interval and aggressive imaging characteristics of the tumor, a malignant tumor was suspected and microsurgical resection was arranged.

A right retrosigmoid approach was selected for tumor resection. After identifying the lower cranial nerve group, a lesion within the CPA was identified, light brown in color, with a soft consistency. Tumor was debulked in a piecemeal fashion. Due to multiple infiltrative regions to the adjacent pons, GTR could not be safely performed and STR was completed instead.

Histopathology revealed features consistent with the glioblastoma. Staining was immunopositive for Olig2, with CD56, Synaptophysin, NSE, and S100 positive stroma (Supplementary Fig. 3). Staining for Vimentin, EMA, and CKAE1/AE3 were negative. The postoperative course was uneventful, and the patient received institutional protocol-based adjuvant therapy that included chemotherapy with temozolomide and external beam fractionated irradiation to a total dose of 74 Gy.

## Discussion

Glioblastomas, with an incidence rate of 3.23 per 100,000 population, are the most common malignant tumors of the CNS^1^. Distinctive group of CPA glioblastomas are considered very rare. Given their rarity and peculiar anatomical localization, the prognosis of CPA glioblastomas and factors influencing survival are unclear. A management protocol and clear guidelines for CPA glioblastomas are yet to be defined. Knowledge about the factors associated with survival is essential in guiding neurosurgeons and oncologists in treatment. We, therefore, performed a first comprehensive analysis to identify and determine factors influencing survival in patients with CPA glioblastomas (to the best of our knowledge).

**Figure 5:**
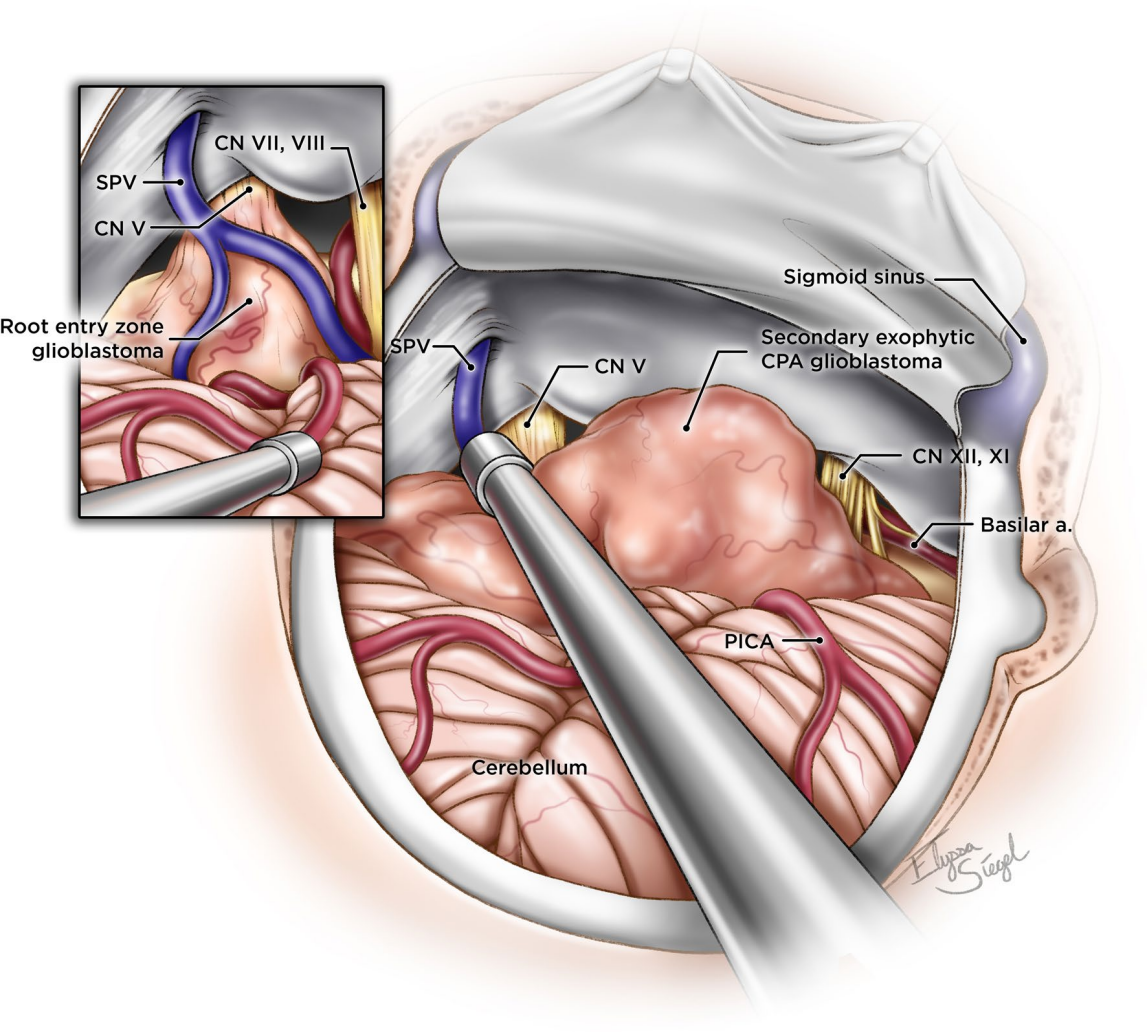
Artist’s illustration of exophytic and nerve REZ (inset) CPA glioblastoma ©Elyssa Siegel 2022.

**Figure 6:**
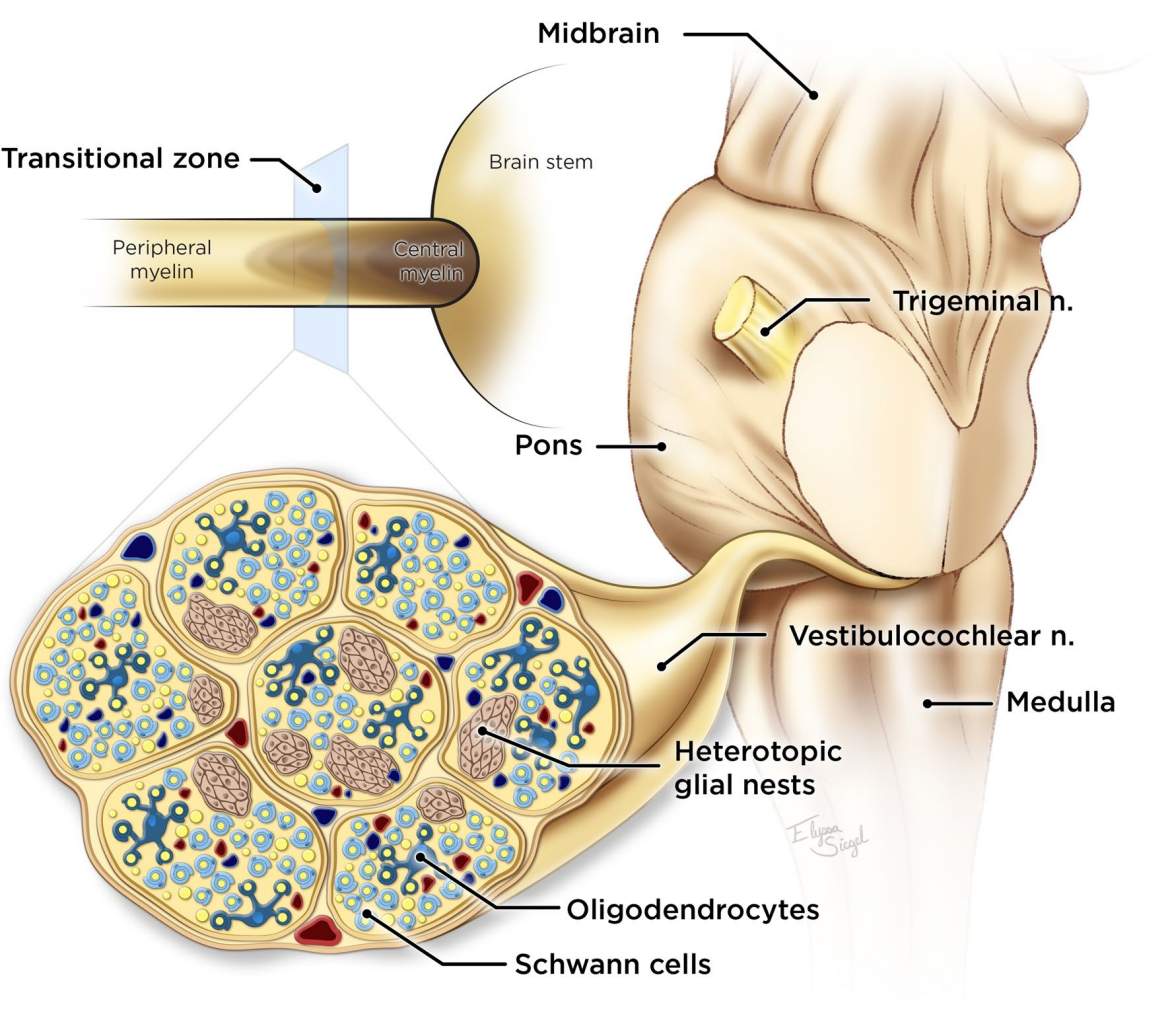
A cross-section of the funnel-shaped transitional zone within the nerve REZ depicts distinct islands of neuroglial tissue, likely the origin of nerve REZ gliomas. In the transitional zone, both Schwann cells and oligodendrocytes are present ©Elyssa Siegel 2022.

Several theories may explain how these tumors develop. They may arise as secondary extensions of tumors that develop in the superficial part of the brainstem (Fig. 5)^8^. Occasional islands of heterotopic glioneural tissue across the CNS, including the leptomeninges, nerve REZ, and the peripheral segment may also be the origin of these tumors (Fig. 6)^5,9,10^. Molecular profiling of the CPA glioblastoma has revealed underlying genetic mutations in the TP53, TERT, NF1, and RB1 genes^11^. Taraszewska et al*.*^12^ reported bilateral CPA glioblastoma in a patient with neurofibromatosis type 1, suggesting a possible genetic link.

The mean age in our cohort was lower when compared with the reported age in the literature, with a slight male predominance, usually observed in glioblastomas^1,7^. Posterior fossa glioblastomas may prove to be a completely different entity. Their histology shows similarities to secondary glioblastomas that occur at an earlier age, considering readily observed absence of EGFR positivity, which may also account for the younger age observed in CPA glioblastomas^13,14^. Previous studies have repeatedly shown that young age is a well-established predictor of long-term survival in patients with glioblastoma^15–17^. Despite minor differences, subgroup analysis stratified by sex and age failed to show any significant influence on the OS.

Our findings suggest that tumor origin do not seem to be influencing OS. However, due to the small cohort, this finding should be interpreted cautiously. Raw data suggest that a difference in mean OS may exist between groups in which pontine origins have the worst mean OS. The proximity of vital structures within the brainstem and a lower tolerance of the posterior fossa to the mass effect are two factors contributing to the markedly different prognosis of the tumors in this region, with the pons showing higher malignant potential in comparison to other structures^18–20^.

The advancement of microsurgical techniques and other technological developments allowed the neurosurgeon to perform an aggressive, albeit safe, resection to achieve maximal cytoreduction, a cornerstone of treatment in patients with newly diagnosed glioblastoma^21^. The causal association between the extent of resection and the prolonged overall and progression-free survival were demonstrated repeatedly in studies, favoring GTR over STR or biopsy when feasible^21–23^. In clinical practice, concerns about injury to the eloquent brain regions, vascular and nerve structures, and subsequent impairment of quality of life make a goal of GTR hard to achieve. Our findings suggest that GTR was possible in only a fraction of patients with CPA glioblastoma, owing to the fact that preservation of cranial nerves and brainstem integrity hinders the radicality of resection in this region. Although no statistical difference was observed, patients with GTR had a longer mean OS compared to patients with STR or biopsy only.

Surgical resection is an important first step in the treatment of glioblastoma. Even with modern advances, multimodal approach with concomitant chemotherapy and radiotherapy remain a standard of care in these patients^7^. Our analysis points out a clear association between adjuvant therapy and OS (log-rank test, p < 0.001), with radiotherapy and concomitant chemotherapy proven to be superior to radiotherapy alone, as demonstrated earlier^24^. It is, however, reasonable to point out that patients who did not undergo treatment may have had too poor functional status. About one-half of patients who did not receive chemotherapy lived 4 months or shorter. This interval may be too short a survival to be eligible for chemotherapy unless given concurrent with radiation, which was not standard before about year 2000. In general, however, the observed mean survival in the CPA angle cohort was 8.9 months, shorter than the 12–14 months reported by Stupp et al*.*^7^.

Posterior fossa tumors are associated with the risk of development of both obstructive and communicating hydrocephalus from as low as 10% up to 50%^25–27^. A nationwide French study of adult patients with cerebellar glioblastoma by Picart et al*.*^28^ reported that 41.5% of patients presented with hydrocephalus, similar to our cohort (36.7% of patients). Unlike cerebellar glioblastoma, hydrocephalus on admission was a negative predictor of OS in patients with CPA glioblastoma. It is difficult to link hydrocephalus directly to OS due to the observed difference in tumor size between groups based on the presence or absence of hydrocephalus.

Despite the fact that no characteristic features on MRI imaging were identified, MR spectroscopy may provide necessary information for suspecting a tumor with glial origins. Avidly enhanced CPA lesions associated with the extension to the IAC and the “choline-only” spectrum most likely suggest VS as the first diagnostic option. Glioblastomas, however, do not present with such a neurobiochemical profile. In glioblastomas, MR spectroscopy typically shows high choline-to-creatine ratio (increased cell membrane turnover), and markedly decreased *N*-acetyl aspartate, a marker of neuronal integrity and function (due to destroyed neuronal tissue, neuronal dysfunction, or rarefaction). The elevated lactate and lipid peaks in glioblastoma are most probably a consequence of anaerobic glycolysis and tumor necrosis, respectively. On the other hand, elevated choline and lipid peaks—with peaks attributable to *N*-acetyl aspartate and creatine being absent—are typical for metastases, while a choline-only spectrum is characteristic of either extraaxial or non-glial intraaxial neoplasms^29,30^. To the best of our knowledge, the illustrative case reported in our study presented with the biochemical profile consistent with that of an extraaxial tumor is also a novelty in the literature.

This particular anatomical region usually harbors benign tumors, including the diagnosis of meningioma and VS. However, rapid neurological and radiological progression with peculiar findings on MR spectroscopy should be considered a red flag and raise suspicion of a potential malignant nature, with CPA glioblastoma as one of the potential diagnoses.

## Conclusion

CPA glioblastomas may arise as exophytic extensions from the adjacent pons or cerebellum, or within the REZ of the cranial nerves. Hydrocephalus on admission in our study was associated with worse OS. Surgical resection followed by adjuvant treatment, provided survival benefit in patients with CPA glioblastoma irrespective of the extent of resection. Retrospective design and small number of available CPA glioblastoma cases included in analysis are inherent limitations of this study. Despite these limitations further studies may provide more data for analysis in the future to determine distinct molecular profiles of the tumors and identify additional factors influencing the survival and management of CPA glioblastomas.

## Methods

### Literature review

#### Literature search

The literature search of PubMed, Web of Science, and Scopus databases was performed according to the Preferred Reporting Items for Systematic Reviews and Meta-Analyses (PRISMA) guidelines^31^. The search was conducted using the strings of search terms, including “glioblastoma”, “glioblastoma multiforme”, and “cerebellopontine angle”. Duplicate results were eliminated and articles were surveyed according to the inclusion criteria described below.

#### Selection criteria

We considered articles published in English and fitting the description of CPA glioblastoma from 1979—when the first reported case was published—to December 2021—when the search was concluded. Two separate reviewers surveyed article titles and abstracts. Full-text articles deemed relevant were examined, and any disagreements were resolved in group discussion. A manual survey of the references within selected articles was performed to reveal references not covered in the original search. Corresponding authors from studies with no individual patient data were contacted by email addresses supplied in each manuscript to provide the missing information.

### Data abstraction

The variables of each patient were extracted and included the following: age, gender, presenting symptoms and duration, presence of hydrocephalus, magnetic resonance imaging (MRI) characteristics, tumor origins, treatment modality, immunohistochemistry, follow-up, and OS.

The extent of surgical resection was classified into gross total resection (GTR), subtotal resection (STR), or biopsy. Adjuvant treatment consisted of radiotherapy or chemotherapy alone, or complete adjuvant therapy with both treatment modalities. OS was measured in months as the time interval from tumor diagnosis until the patient’s death due to any cause. All relevant data are provided within the manuscript and its Supplementary Digital Content files.

### Statistical analysis

The variables were analyzed and charts made using the statistical software package SPSS 21.0 for Windows (IBM Corp. in Armonk, NY). Data were presented using means, ranges, and standard deviations for continuous variables and numbers and proportions for categorical variables. The impact of variables on survival was assessed using the univariate Cox proportional-hazards model to evaluate the association between risk factors and the OS. For significant risk factors, Kaplan–Meier survival curves were used to display survival, and the log-rank test used to compare survival times in each group. Variables that showed statistical significance and trend towards significance (p < 0.1) were included in the final multivariate Cox proportional-hazards model. Hazard ratios (HR) were reported along with 95% confidence intervals (CI). A P-value of 0.05 or less was considered statistically significant.

### Ethical approval

The studies involving human participants were in accordance with the ethical standards of the institutional and/or national research committee and with the 1964 Helsinki Declaration and its later amendments or comparable ethical standards. The study was approved by the institutional ethical review board (Ethical Review Board of the Clinical center of Vojvodina).

## Supplementary Information

Supplementary Figure 1.

Supplementary Figure 2.

Supplementary Figure 3.

Supplementary Table 1.

Supplementary Table 2.

## Data Availability

The original contributions presented in the study are included in the article/Supplementary Material, further inquiries can be directed to the corresponding authors.
