# Supplementary material for: An integrative survival analysis and a systematic review of the cerebellopontine angle glioblastomas
Source: Sci Rep. 2023 Mar 17;13:4442. doi: 10.1038/s41598-023-30677-x (PMC10023706; doi:10.1038/s41598-023-30677-x)
Supplement: Supplementary file 3 — Supplementary Figure 3. [file 41598_2023_30677_MOESM3_ESM.docx]

**An integrative survival analysis and a systematic review of the cerebellopontine angle glioblastomas**

Nebojsa Lasica^1,2^, Kenan Arnautovic^3,4^, Tomita Tadanori^5^, Petar Vulekovic^1,2^, Dusko Kozic^2,6^

^1^Clinic of Neurosurgery, University Clinical Center of Vojvodina, Novi Sad, Serbia

^2^Faculty of Medicine, University of Novi Sad, Novi Sad, Serbia

^3^Semmes Murphey Clinic, Memphis, Tennessee, USA

^4^Department of Neurosurgery, University of Tennessee Health Science Center, Memphis, Tennessee, USA

^5^Division of Pediatric Neurosurgery, Ann & Robert H. Lurie Children’s Hospital of Chicago and Northwestern University Feinberg School of Medicine, Chicago, Illinois, USA

^6^Center for Diagnostic Imaging, Oncology Institute of Vojvodina, Sremska Kamenica, Serbia

**CORRESPONDING AUTHOR**

Nebojsa Lasica

Email: nebojsa.lasica@mf.uns.ac.rs

Clinic of Neurosurgery, University Clinical Center of Vojvodina

Hajduk Veljkova 1-9, 21000 Novi Sad, Serbia

Telephone: +381 64 381 0644

**Supplementary Figure 3.** Photomicrographs of pathological specimens. (**A**) A tumor composed of astrocytic cells and collagenous stroma. H&E. Original magnification x100. Immunohistochemistry showed Olig2 (**B**), CD56 (**C**), Synaptophysin (**D**), NSE (**E**), and S100 (**F**) expression. Original magnification x100 (**B**, **C**), x200 (**D-F**)

**
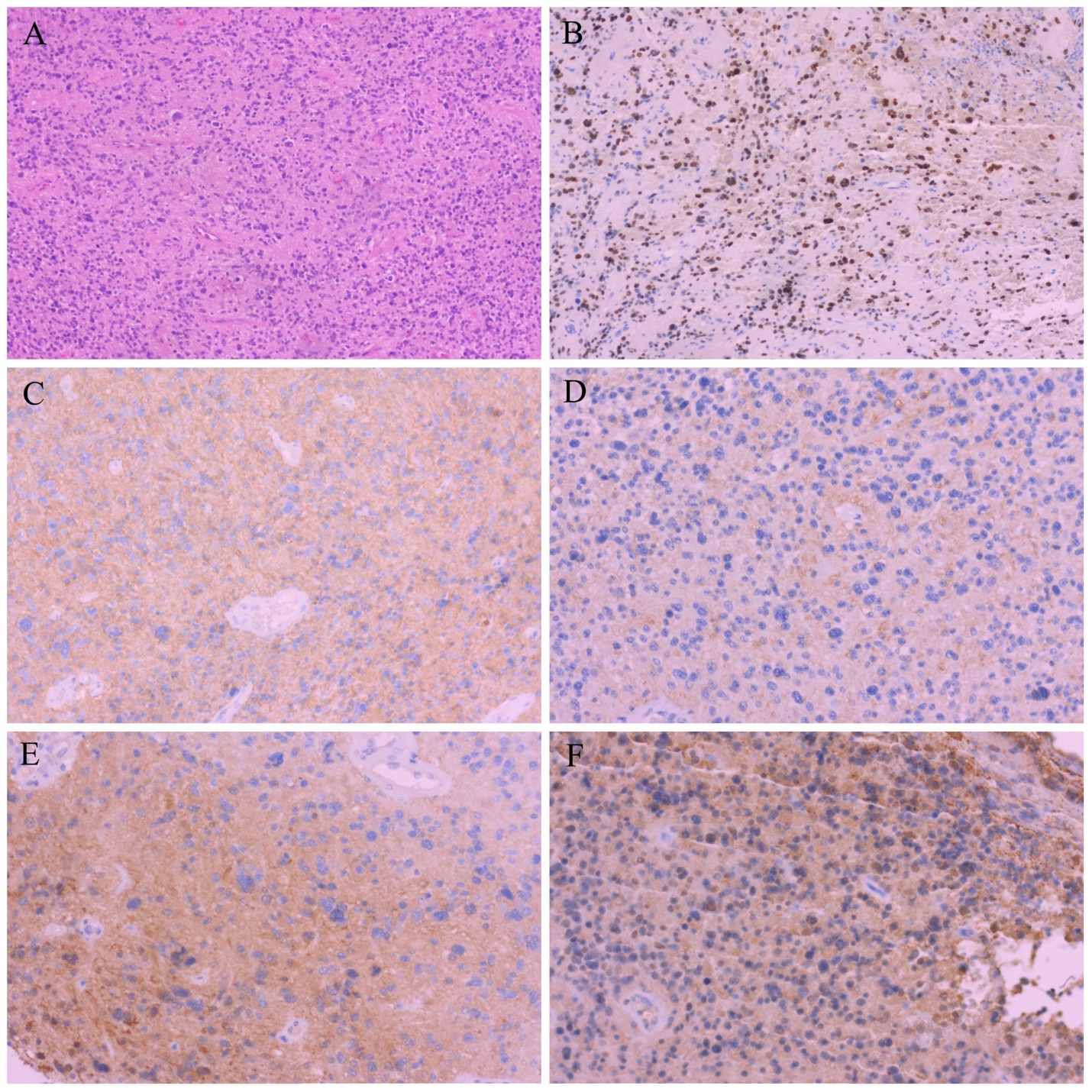
**
