# Supplementary material for: An integrative survival analysis and a systematic review of the cerebellopontine angle glioblastomas
Source: Sci Rep. 2023 Mar 17;13:4442. doi: 10.1038/s41598-023-30677-x (PMC10023706; doi:10.1038/s41598-023-30677-x)
Supplement: Supplementary file 4 — Supplementary Table 1. [file 41598_2023_30677_MOESM4_ESM.docx]

**An integrative survival analysis and a systematic review of the cerebellopontine angle glioblastomas**

Nebojsa Lasica^1,2^, Kenan Arnautovic^3,4^, Tomita Tadanori^5^, Petar Vulekovic^1,2^, Dusko Kozic^2,6^

^1^Clinic of Neurosurgery, University Clinical Center of Vojvodina, Novi Sad, Serbia

^2^Faculty of Medicine, University of Novi Sad, Novi Sad, Serbia

^3^Semmes Murphey Clinic, Memphis, Tennessee, USA

^4^Department of Neurosurgery, University of Tennessee Health Science Center, Memphis, Tennessee, USA

^5^Division of Pediatric Neurosurgery, Ann & Robert H. Lurie Children’s Hospital of Chicago and Northwestern University Feinberg School of Medicine, Chicago, Illinois, USA

^6^Center for Diagnostic Imaging, Oncology Institute of Vojvodina, Sremska Kamenica, Serbia

**CORRESPONDING AUTHOR**

Nebojsa Lasica

Email: nebojsa.lasica@mf.uns.ac.rs

Clinic of Neurosurgery, University Clinical Center of Vojvodina

Hajduk Veljkova 1-9, 21000 Novi Sad, Serbia

Telephone: +381 64 381 0644

**Supplementary Table 1.** List of CPA glioblastomas described in the literature.

|  | | | | | | | |
| --- | --- | --- | --- | --- | --- | --- | --- |
| **Authors & Year** | **Age (yrs)/ Sex** | **Presentation** | **Symptoms Duration (mos)** | **Hydro-cephalus** | **Origin** | **Treatment** | **FU/OS (mos)** |
| Bussone *et al.*, 1979^1^ | 49/M | Facial weakness, tinnitus | 2 | No | Cerebellum | None | 5 |
| Ahn and Jackler, 1997^2^ | 79/M | Hearing loss, gait disturbance | 3 | Yes | Cerebellum | STR | 0.8 |
| Yamamoto *et al.*, 1997^3^ | 61/F | Gait disturbance, HA/N/V | 1 | Yes | Cerebellum | STR+RT+CT | 12 |
| Swaroop and Whittle, 1997^4^ | 22/M | Ear pain, gait disturbance | 12 | No | Pons | STR+RT | 12 |
| Jindal *et al.*, 2006^5^ | 15/F | Dizziness | 2 | Yes | Cerebellum | GTR+RT | 3 |
| Rasalingam *et al.*, 2008^6^ | 9/M | Double vision, ataxia | 0.5 | Yes | Pons | STR | 2 |
| Kasliwal *et al.*, 2008^7^ | 11 | Gait disturbance | 1.5 | Yes | Cerebellum | Biopsy | 2 |
| Tsai *et al.*, 2009^8^ |  |  |  |  |  |  |  |
| Patient 1 | 11/M | Gait disturbance, facial weakness, dysarthria | 0.4 | Yes | N/A | STR+RT+CT | 1 |
| Patient 2 | 18/F | Double vision | 2 | Yes | N/A | STR+RT | 168 |
| Patient 3 | 8/M | Hemiparesis | 0 | No | N/A | Biopsy+RT | 7 |
| Wu *et al.*, 2011^9^ | 60/M | Facial weakness, hearing loss | 2 | No | VIII | STR | 2 |
| Salunke *et al.*, 2012^10^ | 59/M | Hearing loss | 3 | No | Pons | STR+RT | 9 |
| Taraszewska *et al.*, 2013^11^ | 29/F | HA/N/V | 0 | Yes | Pons | STR | 0.3 |
| Breshears *et al.*, 2015^12^ | 67/M | Facial numbness | 2 | No | V | Biopsy+RT+CT | 5.8 |
| Matsuda *et al.*, 2014^13^ | 69/M | Facial pain | 0 | No | Cerebellum | STR+RT+CT | 24 |
| Mabray *et al.*, 2015^14^ |  |  |  |  |  |  |  |
| Patient 1 | 67/M | Facial numbness | 2 | No | V | Biopsy+RT+CT | 12 |
| Patient 2 | 24/F | Facial numbness and weakness | - | No | V, VII | - | N/A |
| Tomita and Grahovac, 2015^15^ | 3/F | Hemiparesis | 0.75 | No | Pons | GTR+RT+CT | 9 |
| Duan *et al.*, 2016^16^ | 71/F | Dizziness | 4 | No | Cerebellum | STR+RT+CT | 6 |
| Chen *et al.*, 2017^17^ | 5/F | Strabismus | 2 | No | Pons | STR | 2 |
| Panigrahi *et al.*, 2017^18^ | 52/F | Dizziness, blurred vision, gait disturbance | 2 | Yes | Cerebellum | STR+RT | 3 |
| Jhawar *et al.*, 2017^19^ | 12/M | Gait disturbance | 1 | No | Pons | STR+RT | 12 |
| Lee *et al.*, 2017^20^ | 71/F | Gait disturbance | 3 | No | Cerebellum | Biopsy+RT+CT | 12 |
| Yang *et al.*, 2019^21^ | 55/M | Hearing loss, vertigo, gait disturbance | 3 | No | VIII | STR | 2.5 |
| Yoon *et al.*, 2018^22^ | 78/M | Dizziness, gait disturbance | 3 | No | Cerebellum | GTR | 9 |
| Takami *et al.*, 2018^23^ | 55/M | Vertigo, imbalance | 0 | No | VIII | STR+RT+CT | 15 |
| Kaushik *et al.*, 2019^24^ | 14/F | HA/N/V, drowsiness, hemiparesis | 0.5 | Yes | VII, VIII | STR | 2 |
| Kiyofuji *et al.*, 2021^25^ | 45/M | Hoarseness, gait disturbance, swallowing difficulty | 36 | No | Cerebellum | GTR+RT+CT | N/A |
| Pregúntegui-Loayza *et al.*, 2020^26^ | 6 | HA/N/V, gait disturbance | 2 | Yes | Pons | STR+RT+CT | 12 |
| Our case | 53/M | Facial numbness and weakness, hearing loss | 1 | No | VIII | STR+RT+CT | 24 |
| **Abbreviations:** CT=Chemotherapy; FU=follow up; GTR=gross total resection; HA/N/V=headache, nausea, and/or vomiting; N/A=not available; OS=overall survival; RT=Radiotherapy; STR=subtotal resection. | | | | | | | |

**SUPPLEMENTAL DIGITAL CONTENT 1, TABLE REFERENCES**

1. Bussone, G. *et al.* A case of glioblastoma with multiple centers above and below the tentorium. *J. Neurol.* **221,** 187-192 (1979).

2. Ahn, M. S. & Jackler, R. K. Exophytic brain tumors mimicking primary lesions of the cerebellopontine angle. *Laryngoscope* **107,** 466-471 (1997).

3. Yamamoto, M. *et al.* Cerebellar gliomas with exophytic growth —three case reports. *Neurol. Med. Chir.* **37,** 411-415 (1997).

4. Swaroop, G. R. & Whittle, I. R. Exophytic pontine glioblastoma mimicking acoustic neuroma. *J. Neurosurg. Sci.* **41,** 409-411 (1997).

5. Jindal, A. *et al.* Cerebellar glioblastoma multiforme presenting as a cerebellopontine angle mass. *J. Pediatr. Neurosci.* **1,** 21-23 (2006).

6. Rasalingam, K. *et al.* A rare case of paediatric pontine glioblastoma presenting as a cerebellopontine angle otogenic abscess. *Malays. J. Med. Sci.* **15,** 44-48 (2008).

7. Kasliwal, M. K., Gupta, D. K., Mahapatra, A. K. & Sharma, M. C. Multicentric cerebellopontine angle glioblastoma multiforme. *Pediatr. Neurosurg.* **44,** 224-228 (2008).

8. Tsai, M. H. *et al.* Treatment of cerebellopontine angle tumors in children: a single institution's experience. *J. Pediatr. Hematol. Oncol.* **31,** 832-834 (2009).

9. Wu, B., Liu, W., Zhu, H., Feng, H. & Liu, J. Primary glioblastoma of the cerebellopontine angle in adults. *J. Neurosurg.* **114,** 1288-1293 (2011).

10. Salunke, P., Sura, S., Tewari, M. K., Gupta, K. & Khandelwal, N. K. An exophytic brain stem glioblastoma in an elderly presenting as a cerebellopontine angle syndrome. *Br. J. Neurosurg.* **26,** 96-98 (2012).

11. Taraszewska, A., Bogucki, J., Powala, A. & Matyja, E. Giant cell glioblastoma with unique bilateral cerebellopontine angle localization considered as extraaxial tumor growth in a patient with neurofibromatosis type 1. *Clin. Neuropathol.* **32,** 58-65 (2012).

12. Breshears, J. D. *et al.* Primary glioblastoma of the trigeminal nerve root entry zone: case report. *J. Neurosurg.* **122,** 78-81 (2015).

13. Matsuda, M. *et al.* Exophytic cerebellar glioblastoma in the cerebellopontine angle: case report and review of the literature. *J. Neurol. Surg. Rep.* **75,** e67-e72 (2014).

14. Mabray, M. C. *et al.* Direct cranial nerve involvement by gliomas: case series and review of the literature. *Am. J. Neuroradiol.* **36,** 1349-1354 (2015).

15. Tomita, T. & Grahovac, G. Cerebellopontine angle tumors in infants and children. *Child's Nerv. Syst.* **31,** 1739-1750 (2015).

16. Duan, H. *et al.* Gliosarcoma in the cerebellopontine angle with rapid tumor growth and intratumoral hemorrhage. *World Neurosurg.* **92,** 580.e17-580.e21 (2016).

17. Chen, F. *et al.* Progressive multifocal exophytic pontine glioblastoma: a case report with literature review. *Chin. J. Cancer* **36,** 34 (2017).

18. Panigrahi, S., Mishra, S. & Das, S. Primary cerebellopontine angle glioblastoma in an adult. *Asian J. Neurosurg.* **12,** 62-64 (2017).

19. Jhawar, S., Sarvpreet, G. & Shadangi, T. Exophytic pontine glioblastoma multiforme presenting as cerebellopontine angle mass. *Asian J. Neurosurg.* **12,** 302-304 (2017).

20. Lee, J. H., Kim, J. H. & Kwon, T. H. Primary glioblastoma of the cerebellopontine angle : case report and review of the literature. *J. Korean Neurosurg. Soc.* **60,** 380-384 (2017).

21. Yang, D. X. *et al.* Primary glioblastoma of cerebellopontine angle in adult mimicking acoustic neuroma. *World Neurosurg.* **122,** 48-52 (2019).

22. Yoon, G. Y., Oh, H. J., Oh, J. S., Yoon, S. M. & Bae, H. G. Gliosarcoma of cerebello-pontine angle: a case report and review of the literature. *Brain Tumor Res. Treat.* **6,** 78-81 (2018).

23. Takami, H. *et al.* Glioblastoma of the cerebellopontine angle and internal auditory canal mimicking a peripheral nerve sheath tumor: case report. *J. Neurosurg.* **131,** 1835-1839 (2018).

24. Kaushik, K. & Pandey, S. Primary cerebellopontine angle glioblastoma in a child. A rare entity. *Rom. Neurosurg.* **33,** 49-51 (2019).

25. Kiyofuji, S., Graffeo, C. S., Perry, A. & Link, M. J. Far lateral approach for malignant, severely calcified cerebellopontine angle tumor. *J. Neurol. Surg. B Skull Base* **82,** S29-S30 (2021).

26. Pregúntegui-Loayza, I., Céspedes, E. & Saal-Zapata, G. Glioblastoma multiforme in the cerebellopontine angle in a pediatric patient. *Indian J. Neurosurg.* **10,** 155-158 (2020).
