# Supplementary material for: An integrative survival analysis and a systematic review of the cerebellopontine angle glioblastomas
Source: Sci Rep. 2023 Mar 17;13:4442. doi: 10.1038/s41598-023-30677-x (PMC10023706; doi:10.1038/s41598-023-30677-x)
Supplement: Supplementary file 5 — Supplementary Table 2. [file 41598_2023_30677_MOESM5_ESM.docx]

**An integrative survival analysis and a systematic review of the cerebellopontine angle glioblastomas**

Nebojsa Lasica^1,2^, Kenan Arnautovic^3,4^, Tomita Tadanori^5^, Petar Vulekovic^1,2^, Dusko Kozic^2,6^

^1^Clinic of Neurosurgery, University Clinical Center of Vojvodina, Novi Sad, Serbia

^2^Faculty of Medicine, University of Novi Sad, Novi Sad, Serbia

^3^Semmes Murphey Clinic, Memphis, Tennessee, USA

^4^Department of Neurosurgery, University of Tennessee Health Science Center, Memphis, Tennessee, USA

^5^Division of Pediatric Neurosurgery, Ann & Robert H. Lurie Children’s Hospital of Chicago and Northwestern University Feinberg School of Medicine, Chicago, Illinois, USA

^6^Center for Diagnostic Imaging, Oncology Institute of Vojvodina, Sremska Kamenica, Serbia

**CORRESPONDING AUTHOR**

Nebojsa Lasica

Email: nebojsa.lasica@mf.uns.ac.rs

Clinic of Neurosurgery, University Clinical Center of Vojvodina

Hajduk Veljkova 1-9, 21000 Novi Sad, Serbia

Telephone: +381 64 381 0644

**Supplementary Table 2.** Immunohistochemical characteristics of the CPA glioblastomas

|  | | |
| --- | --- | --- |
| **Variable** | **Tumor origins** | |
|  | **Secondary exophytic (n=19)** | **Nerve root entry zone (n=9)** |
| Immunohistochemistry, n (%) |  |  |
| Immunohistochemistry available |  |  |
| GFAP+ | 11 (57.9) | 4 (44.4) |
| S100+ | 3 (15.8) | 3 (33.3) |
| p53+ | 4 (21.0) | 2 (22.2) |
| Olig2+ | 0 (0.0) | 2 (22.2) |
| Synaptophysin | 0 (0.0) | 1 (11.1) |
| H3 K27me3+ | 0 (0.0) | 1 (11.1) |
| MGMT+ | 2 (10.5) | 0 (0.0) |
| Vimentin+ | 2 (10.5) | 0 (0.0) |
| EGFR+ | 1 (5.3) | 0 (0.0) |
| IDH1+ | 1 (5.3) | 0 (0.0) |
| Desmin+ | 1 (5.3) | 0 (0.0) |
| Immunohistochemistry not available | 7 (36.8) | 2 (22.2) |

**Abbreviations:** EGFR=Epidermal growth factor receptor; GFAP=Glial fibrillary acidic protein; IDH1=Isocitrate dehydrogenase 1; MGMT=O^6^-methylguanine-DNA methyltransferase; Olig2=Oligodendrocyte transcription factor 2.
